# Clinical review of tularemia cases, evaluation of treatment approaches: single-center retrospective study

**DOI:** 10.1007/s00405-026-10261-5

**Published:** 2026-04-23

**Authors:** Murat Demir, Şeyma Betül Kayhan Güzel

**Affiliations:** 1 Department of Otorhinolaryngology, Sakarya University Training and Research Hospital, Sakarya, Turkey; 2 Department of Infectious Diseases and Clinical Microbiology, Lokman Hekim University, Ankara, Turkey

**Keywords:** Tularemia, *Francisella tularensis*, Oropharyngeal tularemia, Oculoglandular tularemia, Cervical lymphadenopathy

## Abstract

**Purpose:**

Tularemia is a zoonotic disease caused by *Francisella tularensis*, a gram-negative, facultative coccobacillus. Tularemia may present with symptoms of tonsillopharyngitis, such as sore throat and neck swelling, or with symptoms of conjunctivitis, such as redness in the eyes. The aim is to emphasize the importance of considering tularemia in the differential diagnosis of patients presenting with these complaints.

**Methods:**

The study was designed as a retrospective descriptive study including 10 patients who were diagnosed with tularemia and treated between November 2023 and August 2025.

**Results:**

The patients were between 19 and 69 years of age, with a mean age of 47.2 ± 18.4 years. Six patients were male and four were female. The most common presenting symptoms were sore throat (*n* = 7, 70%) and cervical lymphadenopathy/swelling (*n* = 7, 70%). The oropharyngeal form was observed in six patients, the oculoglandular form in three patients, and a combination form in one patient. All patients were initially started on doxycycline therapy. In addition to medical treatment, abscess drainage was performed in six patients (60%), and these patients were subsequently started on one of second-line treatments: streptomycin, ciprofloxacin, or gentamicin.

**Conclusion:**

Tularemia often presents with non-specific, influenza-like symptoms and signs of conjunctivitis may therefore be easily overlooked in clinical practice. Clinicians should be well acquainted with the various clinical forms of tularemia and consistently consider the disease in the differential diagnosis. Successful management of tularemia depends on early recognition, the appropriate antimicrobial agents, and the timely implementation of necessary surgical interventions.

## Introduction

Tularemia was first described in 1911 by McCoy as a plague-like disease observed in squirrels in the Tulare region of California, and was initially named *Bacterium tularense* after the area in which it was identified. In recognition of the contributions of Edward Francis, the scientist who first described the disease in humans, the bacterium was later renamed *Francisella tularensis* [1].

Tularemia is a zoonotic infectious disease that occurs in the Northern Hemisphere. Between 1920 and 1960, numerous cases were reported in the United States and the Soviet Union; however, in recent years, the annual number of reported cases in these countries has not exceeded a few hundred, with most cases being sporadic and isolated. In contrast, several European countries have experienced more frequent large-scale outbreaks in recent decades. In particular, the incidence of tularemia has increased in Finland and Sweden in recent years [1, 2]. Moreover, tularemia outbreaks have increasingly been reported over the past decades in previously non-endemic countries such as Turkey, Kosovo, and Spain [3].

*F. tularensis* is transmitted to humans through contact with infected wild animals (especially rabbits and small rodents), arthropod bites (mainly Ixodidae ticks and mosquitoes), inhalation of infected aerosols, consumption of contaminated water or food, and swimming and contact with contaminated hydrotelluric environments. After a short incubation period (3–5 days, up to a maximum of two weeks), infected individuals develop influenza-like symptoms. Depending on the route of entry of *F. tularensis* into the body, tularemia can manifest in humans in six distinct clinical forms. The main clinical forms are oropharyngeal, oculoglandular, typhoidal, pneumonic, glandular, and ulceroglandular tularemia. Lymphadenopathy (LAP) is among the most common clinical findings in the oropharyngeal, oculoglandular, glandular, and ulceroglandular forms [4].

Tularemia may present with diverse and complex clinical manifestations. It is most commonly a chronic infection, and its management often requires multiple courses of different antibiotics in combination with surgical intervention. Early diagnosis of tularemia and improvement of treatment strategies are of critical importance for achieving therapeutic success [5].

The aim of this study was to present the clinical evaluation and management approaches of patients who were followed with a diagnosis of tularemia.

## Methods

This study was conducted with the approval of the Lokman Hekim University Ethics Committee, dated 03.10.2025 and coded 2,025,238. It was designed as a retrospective descriptive study.

The study population consisted of 10 patients who were diagnosed with tularemia and treated between November 2023 and August 2025 following admission to the Otorhinolaryngology and Infectious Diseases outpatient clinics of Bayburt State Hospital. All patients who presented to the otorhinolaryngology or infectious diseases outpatient clinics within the specified dates and were treated with a diagnosis or preliminary diagnosis of tularemia were included in the study. Patients who did not attend regular follow-up visits until completion of treatment, those with missing or insufficient study data, and patients under 18 years of age were excluded from the study. Patients’ presenting complaints, medical history, personal history, and family history were obtained, and a detailed physical examination was performed. Laboratory investigations, including complete blood count, biochemical parameters, and C-reactive protein (CRP), were requested for all patients. The results are presented as frequencies and percentages.

For patients in whom tularemia was suspected at the initial presentation, a microagglutination test was also requested. In addition, patients who were initially treated with a preliminary diagnosis of tonsillitis or cervical LAP (e.g., amoxicillin, ampicillin–sulbactam) but showed no clinical improvement were subsequently evaluated for suspected tularemia, and a microagglutination test was requested. In the microagglutination test, antibody titers greater than 1/128 were accepted as diagnostic criteria for tularemia with the presence of compatible disease. It is important to note that agglutination test titers are typically negative during the first two weeks of illness. In most patients, test positivity may be observed two weeks after the onset of symptoms, with peak titers occurring around four to five weeks [6]. Therefore, an initially negative serological result does not exclude tularemia infection.

Patients presenting with sore throat, tonsillopharyngitis, and cervical LAP were classified as the oropharyngeal form. Conversely, patients exhibiting conjunctivitis along with preauricular or cervical LAP were categorized as the oculoglandular form [6]. If a patient had both tonsillopharyngitis and conjunctivitis along with cervical LAP, they were classified as a combination form [7].

Neck ultrasonography was used for the evaluation of superficially located cervical swellings and LAP, whereas contrast-enhanced neck computed tomography was performed in cases with deeply located suppurative LAP. For antibiotic therapy, the Centers for Disease Control and Prevention (CDC) and World Health Organization (WHO) recommended aminoglycosides as the drug of choice for severe tularemia for 7–10 days. For mild and moderate disease, doxycycline, ciprofloxacin, and gentamicin can be used, but since doxycycline is bacteriostatic for *F. tularensis*, longer treatment courses are recommended. If abscessed lymph nodes are present, drainage is conducted under either local or general anesthesia, depending on the abscess’s location [6].

## Results

The patients were between 19 and 69 years of age, with a mean age of 47.2 ± 18.4 years. Six patients were male and four were female. The demographic characteristics and presenting symptoms of the patients, the clinical histories, radiological findings, and treatment protocols of the patients are systematically summarized (Table 1). The most common presenting symptoms at admission were sore throat (*n* = 7, 70%) and cervical LAP/swelling (*n* = 7, 70%).

**Table 1:** The clinical histories, radiological findings, and treatment protocols of the patients

Patients	Age/sex	Dominant clinical type	Dominant source of infection	Patient’s complaint	Physical examination findings	Medications before diagnosis of tularemia	Imaging	Tularemia diagnosis	Medical treatment	Surgical treatment
Patient 1	68, male	Oropharyngeal	Spring water	Sore throat, fever, dysphagia	Tonsillar hypertrophy and exudation, soft palate and uvula edema, trismus	Amoxicillin-clavulanic acid (7 days), ampicillin-sulbactam and clindamycin (5 days)	Neck CT: Peritonsillar, parapharyngeal edema, no cervical LAP	Micro-agglutination (1/320 positive)	Doxycycline (2 × 100 mg, oral, 14 days)	-
Patient 2	45, male	Combination (Oropharyngeal + oculoglandular)	Spring water	Redness in the right eye, sore throat, fever, headache, myalgia	Tonsillar hypertrophy and exudation, soft palate and uvula edema, chemosis	Amoxicillin-clavulanic acid (4 days) Netilmicin + dexamethasone (7 days, ophthalmic)	Neck US: Reactive LAP measuring 11 × 32 mm at level 1 on the right neck and 18 × 30 mm at level 1 on the left neck	Micro-agglutination (negative)	Doxycycline (2 × 100 mg, oral, 14 days)	-
Patient 3	22, female	Oropharyngeal	Spring water	Sore throat, dysphagia, swelling in the right neck	Right cervical LAP, edema and hyperemia in the right peritonsillar space, leftward displacement of the uvula	Ampicillin-sulbactam (4 × 2 g, 10 days)	Neck CT: On the right, a peritonsillar abscess with a diameter of 25 mm. On the right, numerous LAPs with pathological features, the largest of which is at level 2, measuring 28 × 35 mm	Micro-agglutination (1/1280 positive)	Doxycycline (2 × 100 mg, oral, 14 days), ciprofloxacin (2 × 750 mg, oral, 7 days)	Incisional drainage of peritonsillar abscess
Patient 4	36, female	Oropharyngeal	Spring water	Sore throat, dysphagia, swelling in the right neck	Tonsillar hypertrophy and exudation, right upper cervical and submandibular LAP	Depocillin 1.2 m IU ( single dose), cefdinir (7 days)	Neck CT: On the right, levels 1b-2-3, abscessed LAPs, the largest measuring 40 × 12 × 28 mm	Micro-agglutination (1/640 positive)	Doxycycline (2 × 100 mg, oral, 14 days), gentamicin (5 mg/kg/day, iv, 10 days)	Incisional drainage of abscessed LAPs in the operating room
Patient 5	19, female	Oculoglandular	Spring water	Redness in the right eye, swelling in the right neck	Right eye chemosis, right submandibular LAP	Amoxicillin-clavulanic acid (7 days) Netilmicin + dexamethasone (7 days, ophthalmic)	Neck US: An abscessed LAP measuring 25 × 15 mm at the junction of right level 2–3	Micro-agglutination (1/640 positive)	Doxycycline (2 × 100 mg, oral, 14 days)	Incisional drainage of abscessed LAP
				Relapse after 4 months: swelling in the right preauricular area	Abscess in the right preauricular area	-	Neck US: Abscess measuring 5 × 11 mm in the right preauricular area	Micro-agglutination (1/320 positive)	Streptomycin (15 mg/kg/day, im, 20 days)	Incisional drainage of abscessed LAP
Patient 6	66, male	Oropharyngeal	Spring water	Sore throat, fever, myalgia	Tonsillar hypertrophy and exudation, soft palate and uvula edema, submandibular LAP	Amoxicillin-clavulanic acid (7 days)	Neck US: 20 × 7 mm reactive LAP on the right level 2a.	Micro-agglutination (negative)	Doxycycline (2 × 100 mg, oral, 14 days)	-
Patient 7	51, female	Oropharyngeal	Spring water	Sore throat, dysphagia, swelling in the right neck	Hypertrophy and exudation in right tonsil, right cervical LAP	Amoxicillin-clavulanic acid (7 days), ampicillin-sulbactam (7 days)	Neck US: 10 × 26 mm reactive LAP on the right level 3, reactive LAPs on the bilateral level 2	Micro-agglutination (1/320 positive)	Doxycycline (2 × 100 mg, oral, 14 days)	-
Patient 8	69, male	Oropharyngeal	Spring water	Sore throat, dysphagia, swelling in the left neck	Hyperemia in oropharynx and tonsils, left upper cervical LAP	Amoxicillin-clavulanic acid (7 days), Metronidazole 500 mg, 10 days)	Neck US:Abscess measuring 20 × 36 mm in the left level 1b.	Micro-agglutination (1/320 positive)	Doxycycline (2 × 100 mg, oral, 14 days), streptomycin (15 mg/kg/day, im, 10 days)	Incisional drainage of abscessed LAP
Patient 9	59, male	Oculoglandular	Spring water	Redness in the left eye, swelling in the left neck	Hyperemia in oropharynx and tonsils, left preauricular and upper cervical LAP	Amoxicillin-clavulanic acid (7 days)	Neck US: Abscessed LAPs measuring 38 × 25 mm in the left preauricular area and 26 × 20 mm in the left level 2	Micro-agglutination (1/320 positive)	Doxycycline (2x100 mg, oral, 14 days) ciprofloxacin (2x750 mg, oral, 7 days)	Incisional drainage of abscessed LAPs
Patient 10	37, male	Oculoglandular	Spring water	Redness in the left eye, swelling in the left neck	Abscess in the left preauricular, submandibular area	-	Neck US: Left level 2 abscessed LAP measuring 33 × 15 mm + left preauricular area abscessed LAP measuring 14 × 11 mm	Micro-agglutination (1/640 positive)	Doxycycline (2 × 100 mg, oral, 14 days) ciprofloxacin (2 × 750 mg, oral, 7 days)	Incisional drainage of abscessed LAPs

*CT* Computed tomography, *LAP* lymphadenopathy, *US* ultrasound, *iv*: intravenous, *im* intramuscular

All patients had a history of consuming spring water, face washing, or performing ablution. None of the patients had a history of contact with wild rabbits/rodents or tick/fly bites. At the beginning of the outbreak, it was observed that the initial patients, who had similar complaints, shared a common history of performing ablution and consuming water from a spring located near a specific mosque. Later, patients from the same district or neighboring districts who had been in contact with different sources of water continued to arrive. One of the patients had applied because they had household contact with someone who had similar complaints. After discovering this situation, a field investigation was conducted as part of the outbreak analysis. Samples were taken from water storage tanks and some tap waters in the affected districts, but *F. tularensis* could not be isolated. As a second step, the connections between the spring waters and water storage tanks of the affected districts were examined, and the natural spring waters that were thought to be potentially contaminated were identified. Sampling from these points was planned, but it could not be conducted due to the harsh winter conditions.

90% of the patients (*n* = 9) had received beta-lactam group antibiotic therapy prior to admission, while one patient presented without having received any treatment. Imaging studies were performed in all patients: cervical ultrasonography in seven patients and contrast-enhanced cervical computed tomography in three patients. Imaging revealed no pathological LAP in one patient, whereas LAP was detected in nine patients; with lymph node suppuration, and abscess formation in six patients. Cervical LAP was most frequently detected in level II (*n* = 7, 70%), followed by level I (*n* = 4, 40%), level III (*n* = 4, 40%), and preauricular/periparotid (*n* = 3, 30%) regions.

Laboratory results revealed a mean white blood cell (WBC) count of 11.18 × 10³/µL (reference range: 4–12.5 × 10³/µL), neutrophil count of 7.85 × 10³/µL (reference range: 2–7.1 × 10³/µL), and C-reactive protein (CRP) level of 79.8 mg/L (reference range: 0–5 mg/L). The microagglutination test was positive in eight patients. In two patients whose initial test results were negative, clinical response to treatment was observed at follow-up; however, repeat testing could not be performed because the confirmatory laboratory investigations had not yet been finalized. The polymerase chain reaction (PCR) testing could not be performed because it was unavailable.

60% of the patients were classified as having the oropharyngeal form 30% as the oculoglandular form, and one patient was considered to have a combination form. Since all of our cases had mild and moderate disease, doxycycline was selected because of convenience and ease of use. In addition to medical treatment, abscess drainage was performed in all patients (*n* = 6) in whom an abscess was detected by imaging (ultrasound, computed tomography). Four patients with superficial abscessed LAP underwent percutaneous incisional drainage under local anesthesia. In one patient presenting with peritonsillar abscess and intraoral incisional abscess drainage was performed under local anesthesia. In another patient with multiple and deeply located suppurative LAP, the drainage procedure was carried out in the operating room under general anesthesia. These patients were subsequently started on one of the following second-line treatments: streptomycin, ciprofloxacin, or gentamicin, considering the lack of response to treatment with doxycycline.

Clinical cure was achieved in nine patients with the applied treatment regimens. One patient re-presented four months later with recurrent cervical swelling and was considered to have a relapse. This patient underwent abscess drainage and was treated with streptomycin, resulting in a clinical cure with the new treatment regimen.

## Discussion

Tularemia is a zoonotic infectious disease that can be potentially fatal and affects multiple systems in the body. After 1925, it was reported worldwide, and since the 1930s, it has been known in Turkey. In the early years of tularemia’s emergence in Turkey, cases were primarily reported in the Marmara and Western Black Sea regions. However, since 2004, there has been an increase in reported cases from the Central Anatolia and Eastern Anatolia regions. While the ulceroglandular form of tularemia, typically transmitted through direct contact with infected animals or vectors, was the most commonly reported type both worldwide and in the early years in Turkey, recent decades have seen a notable increase in the prevalence of waterborne transmission and the oropharyngeal form [8–11]. Recently, outbreaks have been reported in the provinces of Sivas [12], Erzurum [13], Muş [14], and Çanakkale [15]. Most cases are linked to the consumption or contact with contaminated water, with the oropharyngeal form being the predominant type associated with these outbreaks. Our study also reported similar findings consistent with the outbreaks reported recently. In our study all of the cases were linked to waterborne transmission: direct contact with spring water, face washing, performing ablution, or consumption of spring water.

In reported tularemia cases in other European countries, the prominent form of infection was the ulceroglandular form, while in Turkey the most common presentation was the oropharyngeal form [9]. The most common clinical forms may vary across different regions. Cases presenting with multiple clinical forms (combination form) may also be observed [7]. A rare variation of ulceroglandular disease is the oculoglandular form. This form can result from direct contact with contaminated hands or exposure to contaminated particles [16]. The oculoglandular form is one of the rarest types of tularemia, affecting only 3% to 5% of cases. The clinical presentations are characterized by unilateral conjunctivitis that is painful, purulent, and granulomatous, accompanied by cervical or preauricular LAP [16]. Ophthalmologists are also particularly aware of the presence of non-specific fever and LAP accompanying conjunctivitis cases, especially in regions where tularemia is endemic [17]. In our study, 60% of cases presented with the oropharyngeal form, and 30% showed oculoglandular involvement. One of these cases exhibited findings consistent with both forms. In the outbreak from Sivas, the primary epidemiological exposures included agricultural occupations and animal husbandry. Furthermore, 5.8% of cases involved contact with contaminated natural water, such as rivers and lakes that contained dead animals or their remains [12]. Çakır Kıymaz et al. [12] and Benli et al. [14] reported that most cases are associated with unchlorinated tap water or activities such as swimming or fishing in lakes or rivers. A few years later, Copur and Surme [17] reported two cases of waterborne oculoglandular tularemia in Tatvan, a province neighboring Mus [14]. These cases were linked to a history of swimming in the local lake. In our article, we observed that the rates of ocular involvement exceeded those documented in the existing literature. We hypothesized that these increased incidences were associated with contact with spring water. Documented exposure in some cases to a specific spring water, either for ablution or consumption, supports this hypothesis.

There is no established gold standard for the diagnosis of tularemia. The microagglutination test (MAT), being widespread and easy to perform, is still considered a reference serological method. A microagglutination titer greater than 1:128 confirms the diagnosis of tularemia. The MAT typically becomes positive within 7–14 days. Culture-based diagnosis has low sensitivity, while PCR may be useful for early detection [18]. In a study by Şahin et al., data from 56 tularemia patients were reported, and 17 of these patients were seronegative. The authors noted that these patients were contemporaneous with an outbreak and exhibited clinical features consistent with tularemia; they acknowledged that the MAT may be negative in the early phase and treated the patients with streptomycin [19].

Differential diagnosis is crucial for patients admitted with sore throats or LAP in the cervical or other regions. The oropharyngeal form of tularemia may be mistaken for streptococcal or other non-specific tonsillopharyngitis, while the ulceroglandular form of tularemia can be confused with cat-scratch disease or toxoplasmosis [20]. Oculoglandular tularemia should be differentiated from Parinaud syndrome [21], cat-scratch disease, adenoviral, herpes simplex, or bacterial conjunctivitis. The glandular form may be misdiagnosed as tuberculous LAP, toxoplasmosis, brucellosis, or noninfectious conditions like metastasis, lymphoma, or sarcoidosis [20]. In the case reported by Boeckel et al. [22], a patient who was admitted with solely cervical LAP was differentiated from sarcoidosis, tuberculosis, bartonellosis, toxoplasmosis, and brucellosis. Karabay et al. [23] emphasized that the oropharyngeal tularemia cases were frequently (6.75%) misdiagnosed as tuberculous LAP. Caglı et al. [24] noted that the initial evaluation of early admitted patients of the series focused on malignancies due to related risk factors, and tularemia-related risk factors were overlooked. In our study, some patients were initially treated for tonsillopharyngitis, others misdiagnosed with conjunctivitis, and some evaluated for lymphoma. These patients were treated for tonsillopharyngitis with beta-lactam antibiotics; however, *F. tularensis* can produce beta-lactamase enzymes and become resistant to those antibiotics [5, 25]. Such treatment delays specialist referrals and may lead to complications like lymph node suppuration [24, 26]. In a study by Tezer et al. [25], all 16 patients had beta-lactam antibiotic usage before admission, and 7 of them needed surgical drainage. In our study, 9 out of 10 patients had a history of using beta-lactam antibiotics, including penicillin G, amoxicillin-clavulanate, and ampicillin-sulbactam. All patients who did not respond to beta-lactam treatment were referred to infectious disease or otorhinolaryngology clinics. Imaging studies were conducted for all patients; 7 out of 10 underwent ultrasonography, while 3 out of 10 had computed tomography. While one patient showed no cervical LAP, 9 out of 10 exhibited cervical LAPs, and 6 out of 10 presented with lymph node suppuration and abscess formation. In cases of cervical LAP, Level II was the most commonly affected region among all patients, which is consistent with the case series presented by Caglı et al. [24]. Among the patients treated with doxycycline, six required surgical drainage, whereas four experienced recovery. In the presence of an abscess, in addition to medical treatment, intervention methods such as needle aspiration, drainage, or lymph node excision should be planned. In patients with long-standing and treatment-resistant cervical involvement, super selective cervical dissection and lymph node removal may be necessary [5, 27]. The patients who needed surgical drainage subsequently received a second course of treatment with streptomycin. In tularemia treatment, streptomycin, doxycycline, and tetracycline are the only antimicrobial agents that are currently FDA-approved drugs, and ciprofloxacin and gentamicin have been used off-label for treatment and prophylaxis [28]. According to existing literature, treatment duration is 7–10 days for aminoglycosides and 2–3 weeks for tetracyclines and fluoroquinolones. In some studies, doxycycline is associated with poorer outcomes than other drugs [5]. There is evidence supporting the use of doxycycline as a first-line agent in the treatment of tularemia in most cases, given its long-standing use in the management of human tularemia, strong in vitro and animal data, and the effectiveness observed with early initiation of therapy [27]. These findings show that treatment delay and an unsuitable antimicrobial choice led to treatment failure and complications like suppuration. One patient experienced a relapse four months after therapy, with recurrent swelling on the neck. The patient underwent surgical drainage and received a new course of antibiotic therapy with streptomycin. This patient had close contact with another patient and experienced a delay in admission after the other patient’s treatment course was completed. The relapse may have been linked to the delay in admission and the initial treatment received. The study conducted by Caglı et al. also includes a relapse case, but there is no additional information about the case history. This patient also had a second course of surgical drainage and additional antibiotic therapy with ciprofloxacin [24].

In summary, we have presented a retrospective descriptive study from Bayburt, East Anatolia, and it revealed that while the oropharyngeal form is most common, there is a notably higher rate of the oculoglandular form than previously reported. Waterborne transmission remains the primary route; eye mucous membranes’ direct contact with contaminated water leads to oculoglandular involvement. Our findings emphasized that clinicians should maintain a high index of suspicion for cases of tonsillopharyngitis or conjunctivitis accompanied by LAP, particularly when standard antibiotic therapy is ineffective. Risk factors, including urban residence, living in endemic areas, or exposure to contaminated water, must be assessed. Appropriate antimicrobial therapy should start immediately to reduce complications such as lymph node suppuration and the need for surgical intervention. Increased awareness and consideration of tularemia in differential diagnoses will help improve patient outcomes in endemic regions.
